# Fenbendazole Attenuates Bleomycin-Induced Pulmonary Fibrosis in Mice via Suppression of Fibroblast-to-Myofibroblast Differentiation

**DOI:** 10.3390/ijms232214088

**Published:** 2022-11-15

**Authors:** Lan Wang, Kai Xu, Ningdan Wang, Linke Ding, Wenyu Zhao, Ruyan Wan, Weiming Zhao, Xiaoshu Guo, Xin Pan, Juntang Yang, Ivan Rosas, Guoying Yu

**Affiliations:** 1 State Key Laboratory Cell Differentiation and Regulation, Henan International Joint Laboratory of Pulmonary Fibrosis, Henan Center for Outstanding Overseas Scientists of Pulmonary Fibrosis, College of Life Science, Institute of Biomedical Science, Henan Normal University, Xinxiang 453007, China; 2 Division of Pulmonary, Critical Care and Sleep Medicine, Baylor College of Medicine, Houston, TX 77030, USA

**Keywords:** pulmonary fibrosis, fenbendazole, fibroblasts, glycolysis, AMPK

## Abstract

Idiopathic pulmonary fibrosis (IPF) is a chronic, progressive, and fatal interstitial lung disease with unknown etiology. Despite substantial progress in understanding the pathogenesis of pulmonary fibrosis and drug development, there is still no cure for this devastating disease. Fenbendazole (FBZ) is a benzimidazole compound that is widely used as an anthelmintic agent and recent studies have expanded the scope of its pharmacological effects and application prospect. This study demonstrated that FBZ treatment blunted bleomycin-induced lung fibrosis in mice. In vitro studies showed that FBZ inhibited the proliferation and migration of human embryo lung fibroblasts. Further studies showed that FBZ significantly inhibited glucose consumption, moderated glycolytic metabolism in fibroblasts, thus activated adenosine monophosphate-activated protein kinase (AMPK), and reduced the activation of the mammalian target of rapamycin (mTOR) pathway, thereby inhibiting transforming growth factor-β (TGF-β1)-induced fibroblast-to-myofibroblast differentiation and collagen synthesis. In summary, our data suggested that FBZ has potential as a novel treatment for pulmonary fibrosis.

## 1. Introduction

Idiopathic pulmonary fibrosis (IPF) is a devastating fibrotic interstitial lung disease with a poor prognosis [1,2,3]. It occurs primarily in older adults and is characterized by progressive worsening of dyspnea and lung function [3]. Although the pathogenesis of IPF remains elusive, it is believed to result from an abnormal wound-healing response [4,5]. Recurrent injury to the lung epithelium leads to an abnormal repair process in which various pro-fibrotic cytokines are released causing persistent activation of fibroblasts and other mesenchymal cells and thus disrupting extracellular matrix (ECM) homeostasis [6]. Excessive ECM deposition disturbs the normal architecture of the lung and leads to organ dysfunction and failure and ultimately to death [7]. The potential etiologic factors of IPF include aging, genetics, infection, gastroesophageal reflux, and environmental factors (e.g., cigarette smoke and air pollution) [8,9]. Currently, pirfenidone and nintedanib are the only optional drugs that can retard the progression of the disease [3]. However, there is no effective therapy other than lung transplantation that can reverse the established pulmonary fibrosis [10]. Therefore, finding an effective and affordable new therapy for IPF as well as other fibrotic diseases remains imperative.

In recent years, metabolic dysregulation has been increasingly recognized as a pivotal contributor to the pathogenesis of multiple fibrotic diseases [11,12]. One of the foremost metabolic changes in fibrotic disease is the augmentation of the glycolytic pathway [13]. Glucose is a major source of cellular energy and biosynthetic intermediates required for the production of nucleotides, lipids, and amino acids [14]. During the progression of lung fibrosis, the glycolytic pathway is enhanced to meet both the ATP production and anabolic metabolism need for myofibroblast differentiation and matrix production [15]. In IPF, not only do myofibroblasts prefer aerobic glycolysis as the first source of glucose-derived energy but also glycolysis is required to initiate its differentiation and promote fibrosis progression [12]. Targeting metabolic dysregulation has become an attractive strategy for treating fibrotic diseases [11].

Fenbendazole (FBZ) is a synthetic benzimidazole compound widely used in the treatment of various parasite infections in laboratory animals, livestock, and companion animals [16]. It is characterized by long-lasting efficacy, low side effects, and high tolerance [17]. The anthelmintic activity of FBZ mainly comes from its binding affinity to β-tubulin and disruption of glucose metabolism in intestinal parasite. Recent studies have expanded the scope of its pharmacological effects and gradually revealed previously unrecognized functions [18]. For example, FBZ has been reported to have anti-tumor application prospects due to its highly effective anti-proliferative activity, which mainly comes from its microtubule inhibitory activity and disruption of glucose metabolism [17,19,20,21]. Currently, FBZ is not approved for any clinical use. However, thousands of desperate cancer patients globally have been self-administering the drug, thus raising the need for its preclinical study [22]. In addition, FBZ treatment reduces the production of inflammatory cytokines, such as Interferon gamma (IFN-γ), Tumor necrosis factor alpha (TNF-α), and Interleukin-1 beta (IL-1β), and thus regulates the cellular immune responses of peripheral blood lymphocytes [16,19]. In a mouse model of traumatic spinal cord injury, FBZ treatment improved pathological and functional recovery, suggesting it has a positive role in tissue damage repair [23]. As an anthelmintic commonly used on laboratory animals, FBZ has been shown to affect research outcomes through its pharmacologic mechanism of action. However, to date, no benzimidazole compound has been studied in fibrotic diseases.

In this study, we aimed to determine the role of FBZ in lung fibrosis with a bleomycin-induced mouse model. Our work demonstrated that FBZ attenuated bleomycin-induced mouse lung fibrosis, potently inhibited the viability and mobility of cultured lung fibroblasts, and suppressed TGF-β1-induced fibroblast-to-myofibroblast differentiation. The underlying mechanism may be attributed to the disruption of the microtubule cytoskeleton and moderation of the glycolysis pathway.

## 2. Results

### 2.1. Fenbendazole Attenuated Bleomycin-Induced Lung Fibrosis in Mice

**Figure 1:**
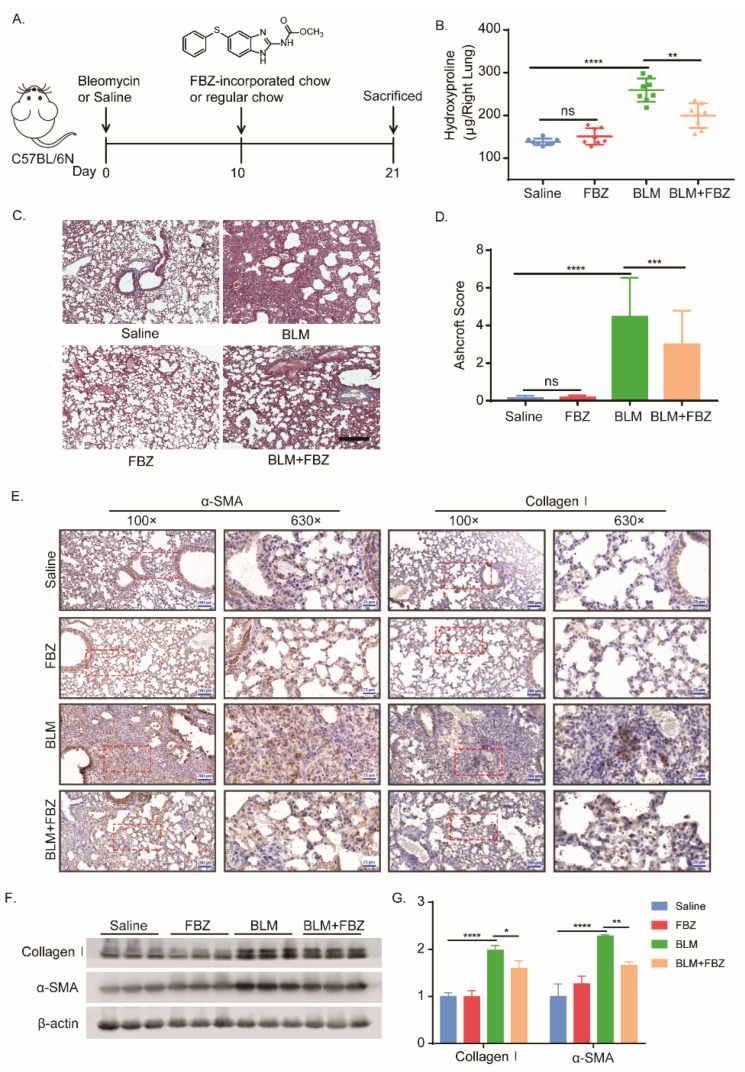
FBZ attenuated bleomycin-induced lung fibrosis. C57BL/6 mice received intratracheal instillation of bleomycin (1.5 U/kg) or equal volume 0.9% saline. Mice were fed with the regular feed (control group) or FBZ-impregnated feed (150 ppm FBZ) from day 10 post bleomycin challenge for additional 10 days. (**A**) Schematic diagram illustrating the time course of treatment with FBZ after bleomycin-induced lung injury. The effect of FBZ on pulmonary fibrosis was assessed by (**B**) quantitative hydroxyproline assay of the right lung (mean ± SD, *n* = 8 mice per group) and confirmed by (**C**) representative images of Masson’s trichrome staining of the lung section (bar = 300 μM) and (**D**) Ashcroft score (mean + SD). (**E**) Representative immunohistochemical stain images of collagen I and α-SMA. (**F**) Immunoblot assay of collagen I and α-SMA in the lung homogenate; β-actin used as the loading control. (**G**) Corresponding optical densitometry analysis of (**F**) (mean + SD). * *p* < 0.05, ** *p* < 0.01, *** *p* < 0.001, and **** *p* < 0.0001.

To investigate the effect of FBZ on fibrogenesis in vivo, the mouse pulmonary fibrosis model was established by single-dose bleomycin administered intratracheally (Figure 1A). No mice died in the experimental groups during the experiment period. The endpoint weight gain of the mice in the bleomycin (BLM) group was significantly lower than that in the saline group; the body weight recovery in the FBZ-treated group was significantly higher than that in the bleomycin model group (Figure S1). According to the daily consumption of the chow, the daily intake dose of each group was calculated, and the average daily intake of FBZ in each group ranged from 21.27 ± 3.55 to 24.84 ± 4.20 mg/kg.bw, with no significant difference observed among the groups (Table S1). The lung hydroxyproline content, as a surrogate of collagen, was reduced significantly in the FBZ-treated groups (Figure 1B). After bleomycin challenge, the FBZ-treated groups showed a decreased distortion of lung architecture, reduced lung collagen deposition as evidenced by Masson stain (Figure 1C), and a decreased Ashcroft score (Figure 1D), while there was no observable lung architecture change between the control and FBZ-only groups. Furthermore, the collagen type I (collagen I) and mesenchymal marker α-smooth muscle actin (α-SMA) was notably reduced in the lung section of the FBZ-treated group (Figure 1E). This observation was further confirmed by immunoblot of the lung lysates (Figure 1F,G). Together, these results showed that treatment with FBZ-impregnated chow blunted the bleomycin-induced mouse lung fibrosis.

### 2.2. Fenbendazole Inhibited Fibroblasts Growth and Mobility 

**Figure 2:**
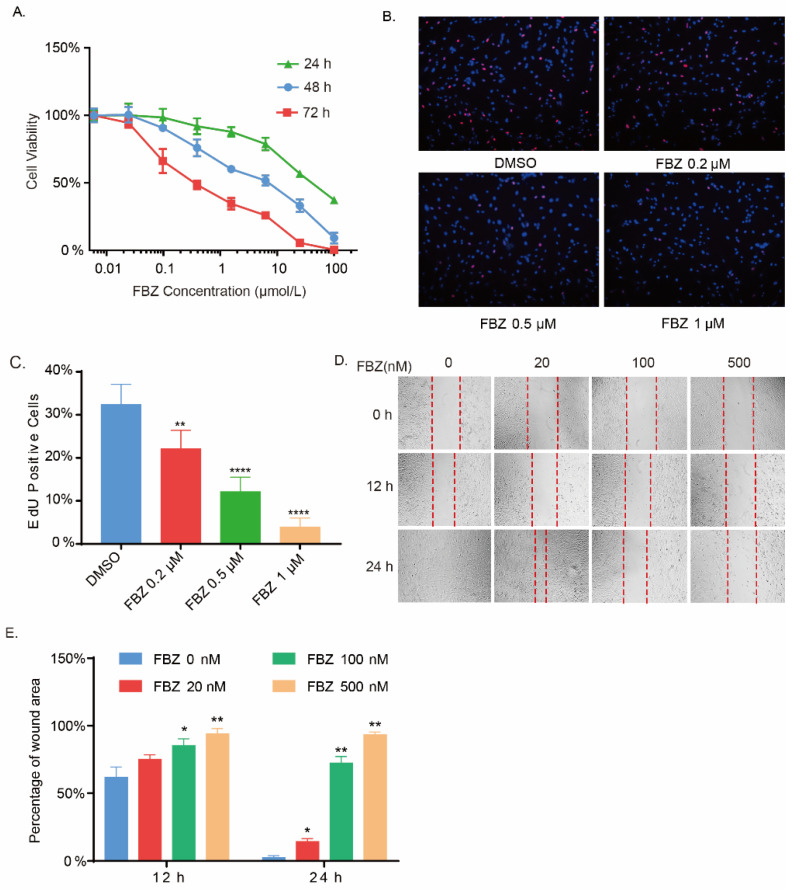
Fenbendazole inhibited fibroblast growth and mobility. (**A**) The concentration–time-dependent inhibitory effect of FBZ on IMR-90 cells was determined by CCK-8 (*n* = 6, mean ± SEM). (**B**) Representative images of EdU assay on FBZ-treated (concentration as indicated) IMR-90 cells; cell nuclei stained with Hoechst are shown in blue pseudocolor, and EdU-positive cell nuclei are shown in red (magnification, 100×). (**C**) Statistics of the EdU-positive cells from (**B**) (*n* = 3, mean + SEM). (**D**) Migration rate of FBZ-treated IMR-90 cells was determined by cell scratch assay. (**E**) Statistics of the wound area from (**D**) (*n* = 3, mean + SEM). * *p* < 0.05, ** *p* < 0.01, and **** *p* < 0.0001.

Lung fibroblasts are the main effector cells of pulmonary fibrosis. Reducing the activation and proliferation of fibroblasts was shown to effectively prevent the development of pulmonary fibrosis [4]. To further explore the anti-fibrosis effect of FBZ, in vitro experiments were assessed on human embryonic lung fibroblast IMR-90 cells. The potential effect of FBZ on the viability and proliferation of fibroblasts was assessed by CCK-8 assay and EdU incorporation assay. FBZ showed a concentration- and time-dependent inhibitory effect on the vitality of fibroblasts (Figure 2A). Consistently, a notable reduction in EdU-positive cells in response to FBZ treatment in a concentration-dependent manner was observed (Figure 2B,C). In addition, FBZ significantly suppressed IMR-90 cell migration evaluated by wound healing assay (Figure 2D,E). Taken together, these results showed that FBZ exhibited potent concentration-dependent inhibitory effects on the proliferation and migration of lung fibroblasts.

### 2.3. Fenbendazole Disrupted Microtubule Cytoskeleton and Caused Cell Cycle Arrest

**Figure 3:**
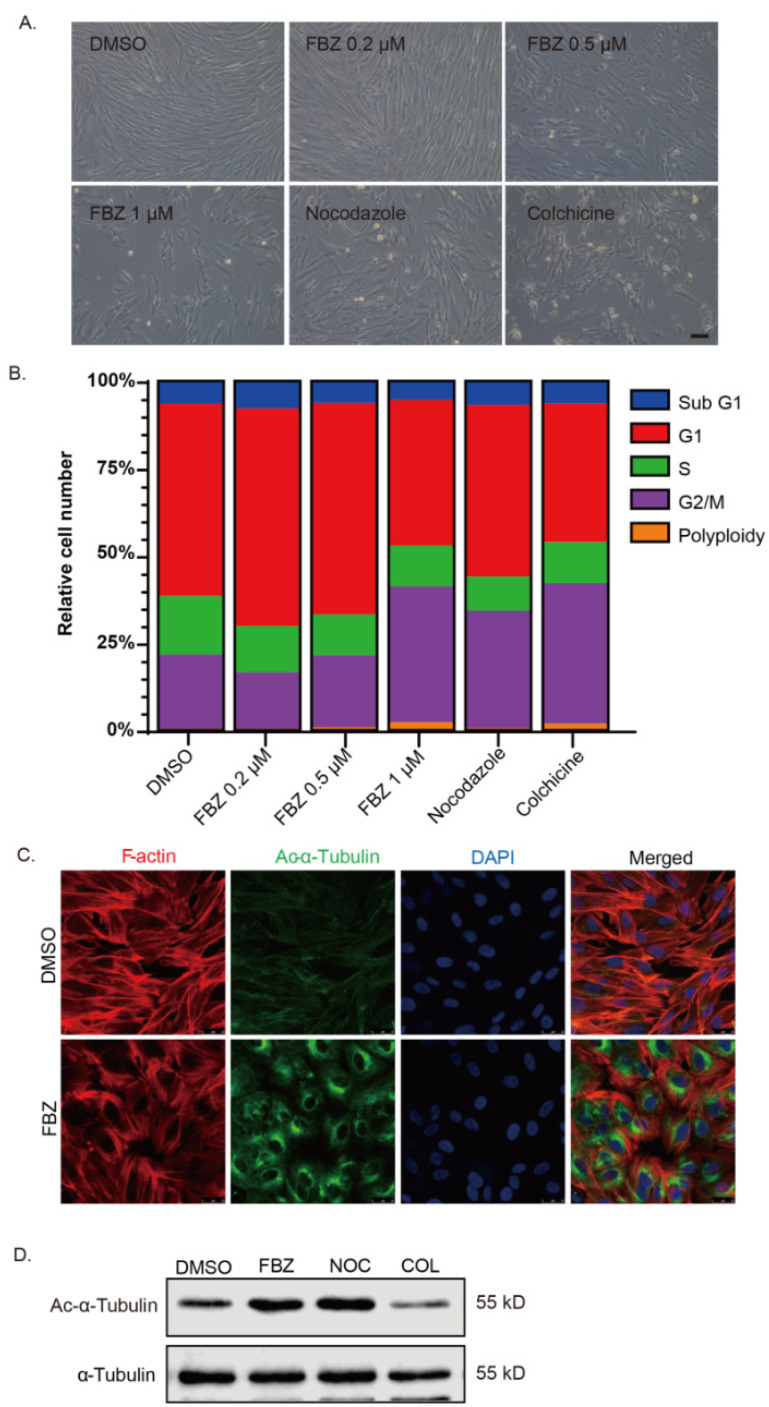
Fenbendazole disrupted microtubule dynamic and caused cell cycle arrest. (**A**) IMR-90 cells were treated with FBZ, nocodazole, colchicine (concentration as indicated), or vehicle for 48 h. Phase-contrast microscopy images showed the morphology of treated cells (magnification 200×). (**B**) IMR-90 cells were treated with indicated reagents for 48 h; alterations in the cell cycle distribution were detected by flow cytometry after PI staining. (**C**) IMR-90 cells were treated with or without 0.5 μM FBZ for 48 h; confocal images were captured after immunofluorescence staining of acetylated α-tubulin (green) and TRITC-conjugated phalloidin (red). (**D**) IMR-90 cells treated with FBZ (0.2 μM), nocodazole (0.1 μM), and colchicine (0.05 μM) for 48 h; acetylated α-tubulin and α-tubulin were assessed by immunoblot.

Previous studies have indicated that FBZ acts as a moderate microtubule-destabilizing agent. We aimed to test if the anti-proliferation effect of FBZ could be attributed to the disruption of the microtubule cytoskeleton. Fibroblasts treated with a high concentration of FBZ (≥0.5 μM) were subject to drastic contractile morphological changes, suggesting disruption of the cytoskeleton (Figure 3A). Consistent with the morphological alteration, a high concentration of FBZ (≥0.5 μM) caused cell cycle arrest in the mitotic phase while it only exhibited a marginal effect at a lower concentration (≤0.2 μM). In comparison, the microtubule inhibitors nocodazole and colchicine caused cell cycle arrest at much lower concentrations (Figure 3B). Immunofluorescent staining showed that the cell morphology changes in the FBZ-treated fibroblasts was associated with reduced stress fiber assembly. However, interestingly, FBZ or nocodazole treatment led to a notable accumulation of acetylated α-tubulin (Figure 3C,D). In comparison, the level of acetylated α-tubulin was significantly reduced in colchicine-treated fibroblasts (Figure 3D). These results indicated that the inhibitory effects on the migration and proliferation of lung fibroblasts could partially be attributed to the disruption of the microtubule cytoskeleton.

### 2.4. Fenbendazole Inhibited Glycolytic Metabolism in Fibroblasts

**Figure 4:**
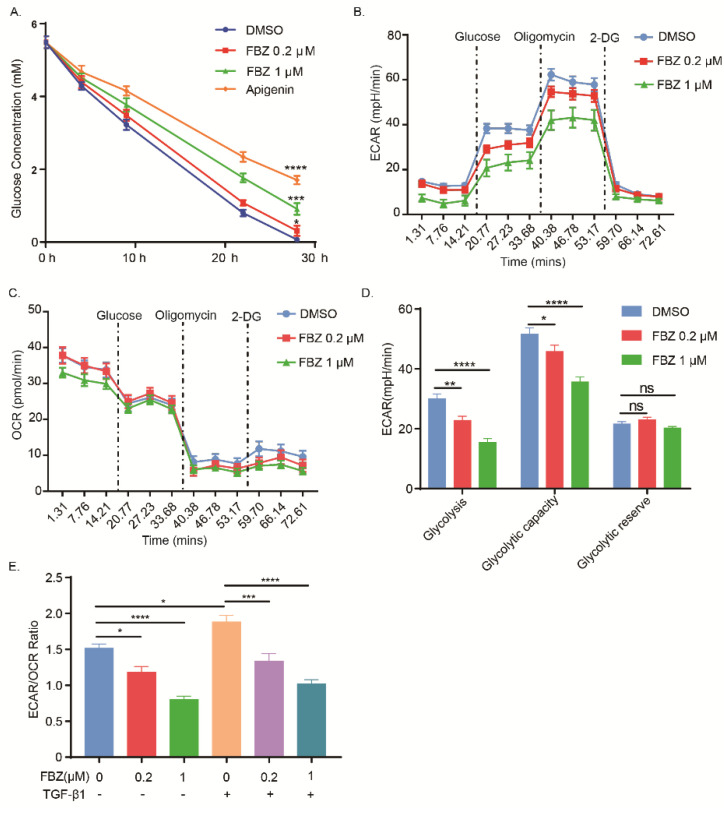
Fenbendazole inhibited glucose metabolism in fibroblasts. (**A**) IMR-90 cells were starved for 2 h in a glucose-free medium before being treated with FBZ (concentrations as indicated) or 50 μM apigenin (as positive control). Glucose concentration in culture supernatants was determined by glucose oxidation assay using a GO assay kit (solarbio) (*n* = 3, mean + SEM). (**B**) Analysis of extracellular acidification rate (ECAR) in IMR-90 cells treated with FBZ at indicated concentrations for 4 h. Cells were treated with glucose, oligomycin A, and 2-Deoxy-D-glucose (2-DG) at indicated time (*n* = 6, mean + SEM). (**C**) Corresponding cellular oxygen consumption rate (OCR) from (**B**). (**D**) ECAR attributed to glycolysis, glycolytic capacity, and the glycolytic reserve was calculated from (**B**). (**E**) Calculated ECAR/OCR ratio in response to glucose addition. * *p* < 0.05, ** *p* < 0.01, *** *p* < 0.001, and **** *p* < 0.0001.

In addition to microtubule destabilizing, benzimidazole anthelmintic has been shown to inhibit glucose utilization with resultant alterations in glucose metabolism. Thus, we aimed to investigate the effects of FBZ on the glucose metabolism of fibroblasts. By measuring concentrations of glucose in culture supernatants, we found that FBZ treatment significantly reduced glucose consumption of cultured fibroblasts (Figure 4A). To investigate the effect of FBZ on glycolysis in fibroblasts, we measured the extracellular acidification rate (ECAR) in cultured lung fibroblasts. ECAR measurement reflects a change in the pH of the assay medium, which is predominantly a result of the glycolysis-mediated lactic acid release after its conversion from pyruvate. Consistent with reduced glucose consumption, the ECAR (Figure 4B) was significantly reduced in FBZ-treated fibroblasts without affecting the corresponding oxygen consumption rate (OCR) (Figure 4C). In particular, FBZ significantly suppressed the glycolysis and glycolytic capacity but not the glycolytic reserve (Figure 4D). Importantly, as TGF-β1 stimulation leads to an augmented ECAR/OCR ratio indicating a shift to aerobic glycolysis, FBZ reduced the ECAR/OCR ratio regardless of TGF-β1 stimulation (Figure 4E).

### 2.5. Fenbendazole Inhibited TGF-β1-Induced Fibroblast-to-Myofibroblast Differentiation and Collagen Formation through AMPK Activation and mTOR Suppression

**Figure 5:**
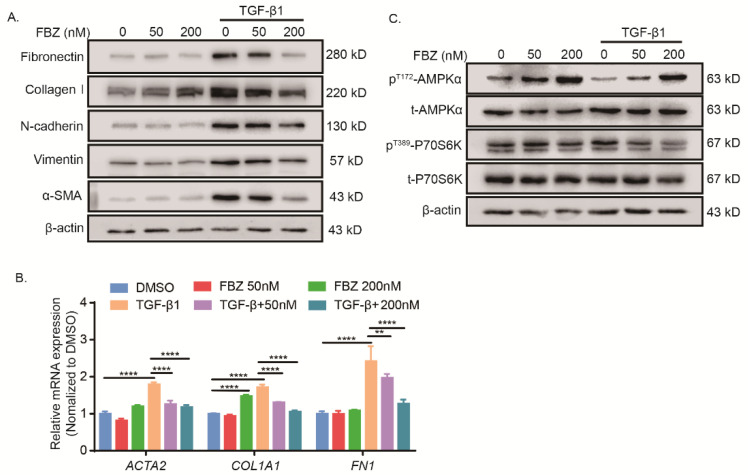
Fenbendazole inhibited TGF-β1-induced fibroblast-to-myofibroblast transition and collagen formation through AMPK activation. (**A**) IMR-90 cells stimulated with or without TGF-β1 for 24 h followed by FBZ treatment (0 nM, 50 nM, or 200 nM) for an additional 24 h. Expression of fibronectin, collagen I, N-cadherin, vimentin, and a-SMA were determined by immunoblot; β-actin used as the loading control. (**B**) RT-qPCR analysis of *ACTA2*, *COL1A1*, and *FN1* mRNA expression in cell lysate treated as (**C**); *ACTB* used as the internal control (*n* = 3, mean + SEM). (**C**) IMR-90 cells treated as (**C**). Phosphorylated (Thr172) AMPKα1/2, total AMPKα1/2, phosphorylated (Thr389) P70S6K, and total P70S6K in cell lysate were determined by immunoblot; β-actin was used as the loading control. ** *p* < 0.01, and **** *p* < 0.0001.

Next, we sought to explore the effect of FBZ on TGF-β1-induced fibroblast activation. FBZ inhibited TGF-β1-induced fibroblast-to-myofibroblast transition, as evidenced by reduced a-SMA and collagen I, as well as fibronectin, N-cadherin, and vimentin, in TGF-β1-stimulated fibroblasts (Figure 5A). This observation was further confirmed by RT-qPCR analysis of *ACTA2*, *COL1A1*, and *FN1* (Figure 5B). To be noted, 200 nM FBZ alone significantly induced expression of *COL1A1* and elevated collagen I but not the other mesenchymal cell markers. Furthermore, FBZ induced AMPK activation by phosphorylation at threonine 172 in fibroblasts regardless of TGF-β1 stimulation (Figure 5C). In addition, TGF-β1-induced mTOR activation was inhibited as phosphorylation of its downstream substrate P70S6K at threonine 389 was down-regulated in response to FBZ treatment (Figure 5C).

## 3. Discussion

In the present study, the effects of FBZ were evaluated in an experimental model of bleomycin-induced lung fibrosis. The results showed that FBZ prevents the progression of bleomycin-induced lung fibrosis, as revealed by preserved lung architecture and decreased ECM deposition. The in vitro results further demonstrated a phenotypical reversion-like phenomenon in TGF-β1-stimulated lung fibroblasts upon FBZ treatment.

Although the etiology of IPF is unknown, it is believed to result from the abnormal wound-healing response after injury. The defining feature of IPF is the accumulation of myofibroblasts, the primary effector cells in fibrogenesis, responsible for the deposition of extracellular matrix and progressive impairment of gas exchange due to the replacement of alveoli with fibrotic tissue [14]. In the in vitro study, the results of the CCK-8 and EdU incorporation assays demonstrated that FBZ inhibited the viability and proliferation of fibroblasts in a concentration- and time-dependent manner. Moreover, FBZ inhibited the cell mobility of fibroblasts potently. The anthelmintic activity of FBZ alongside other benzimidazole compounds mainly comes from their tubulin binding ability which prevents microtubule polymerization. Despite a lower affinity than helminth tubulin, benzimidazole was shown to bind mammalian tubulin [20]. As our data showed, high concentrations of FBZ altered the morphology of the fibroblasts and caused cell cycle arrest in the G2/M phase. Consistent with the observation by Dogra et al. [20], FBZ exhibited a relatively mild effect on mammalian tubulin compared with nocodazole and colchicine. Interestingly, our data showed that a long period of FBZ or nocodazole treatment led to an accumulation of acetylated α-tubulin at lysine 40, unlike colchicine which notably reduced the level of acetylated α-tubulin in cultured fibroblasts. A possible explanation is that those reagents have distinct binding sites and binding affinity on β-tubulin [24], thus causing their different effects on the accumulation of acetylated microtubules. Yet, research regarding the role of acetylated α-tubulin in fibrotic diseases or fibroblast activation has yielded inconsistent conclusions [25,26,27]. For example, nuclear localization of yes-associated protein (YAP) via microtubule acetylation is required for TGF-β1-induced fibroblast-to-myofibroblast differentiation of mouse embryonic fibroblasts cultured on a soft matrix [25]. However, tetra peptide N-acetyl-seryl-aspartyl-lysyl-proline (Ac-SDKP) attenuated silicosis-induced rat lung fibrosis and blocked AngII-induced myofibroblast differentiation by restoring acetylated α-tubulin, suggesting a positive role of acetylated α-tubulin in pulmonary fibrosis treatment [28].

During the progression of fibrosis, lung fibroblasts undergo dramatic metabolic reprogramming in response to activation, thereby promoting proliferation, migration, and ECM synthetic activities [29] One hallmark feature of these metabolic reprogramming events is the up-regulation in aerobic glycolysis. A recent study showed that pharmacologic inhibition of GLUT1-dependent glycolysis provides a potential target of therapy for age-driven fibrotic diseases [30,31]. FBZ, along with some of the other benzimidazole anthelmintics, have been shown to inhibit glucose uptake with resultant alterations in glucose metabolism and ATP production [32,33,34]. Disruption of the microtubule dynamic could lead to the suppression of glucose uptake. However, FBZ may also inhibit glycolysis by directly binding HK II [20]. In these contexts, we sought to understand the role of FBZ in the glycolytic reprogramming of activated fibroblasts. ECAR measurements reflect a change in the pH of the assay medium, which is predominantly a result of the glycolysis-mediated lactic acid release after its conversion from pyruvate. We demonstrated that FBZ inhibited glucose consumption and resulted in the reduction in ECAR in cultured fibroblasts. Most importantly, not only did FBZ reduce the glycolysis and glycolytic capacity, it reduced the ECAR/OCR ratio. An increase in ECAR/OCR ratio in fibroblasts in response to TGF-β1 stimulation indicated a shift to aerobic glycolysis. FBZ treatment reduces glucose consumption and ECAR without affecting OCR, indicating its potent inhibitory effect on aerobic glycolysis.

The anabolism of collagen, as well as other ECM contents, relies on ATP produced by both glycolysis and oxidative phosphorylation, and down-regulation of cellular ATP production has been shown to inhibit collagen secretion [20]. AMPK is an evolutionarily conserved signaling molecule serving as a cellular energy sensor and a master regulator of cellular metabolism for the maintenance of energy homeostasis [35]. AMPK is activated upon various cellular stresses, such as nutrition depletion, hypoxia, and heat shock. In IPF lungs, AMPK activity is largely deficient in α-SMA-expressing myofibroblasts and within regions of active fibrosis [35]. Since FBZ inhibited cellular aerobic glycolysis, we assessed whether the AMPK pathway was affected. Our data showed that FBZ induced AMPK activation and decreased collagen production in TGF-β1-stimulated fibroblasts. These findings were in agreement with those which showed that AMPK activation by pharmaceutical drugs suppressed the TGF-β1-induced collagen production in lung fibroblasts and protected against bleomycin-induced mouse lung fibrosis [30,35,36]. The mTOR pathway is another master regulator of cellular metabolism which is tightly interlinked with AMPK and considered an opposing signaling pathway involved in sensing the availability of nutrients and energy [37,38]. Pharmacological targeting of the mTOR pathway by small-molecule inhibitors such as rapamycin was shown to alleviate experimental pulmonary fibrosis [39,40,41]. Our data showed that exaggerated mTOR activation by TGF-β1 was inhibited by FBZ as evidenced by the reduced phosphorylation of its downstream substrate P70S6K. The interplay of AMPK and the mTOR pathway was tightly linked with glycolytic metabolism, with the availability of glucose acting as a gatekeeper prompting a switch between anabolic and catabolic metabolism [42,43]. In this case, that FBZ treatment resulted in AMPK activation and mTOR inhibition in TGF-β1-stimulated fibroblasts may be attributed to the suppression of glycolysis.

## 4. Material and Methods

### 4.1. Reagents and Antibodies

Recombinant human TGF-β1 was purchased from R&D systems (Minneapolis, MN, USA). FBZ was purchased from Wuxi AppTec (Wuxi, China). Nocodazole and colchicine were purchased from MCE (Shanghai, China). Bleomycin sulfate was purchased from Hisun Pharm (Taizhou, China). Antibody to collagen I, Fibronectin, N-cadherin, phosphor-Thr172 AMPK, AMPKα1/2, phosphor-Thr389 P70S6K, P70S6K, α-Tubulin, acetylated (K40) α-Tubulin, and Alexa Fluor^®^ 488-conjugated anti-rabbit IgG were obtained from Cell Signaling Technology (Shanghai, China). Goat anti-rabbit and anti-mouse IgG with horseradish peroxidase (HRP) conjugate as well as an antibody to α-SMA, β-actin, and Vimentin were obtained from Affinity Biosciences (Changzhou, China).

### 4.2. Mice and the Mouse Model of Bleomycin-Induced Lung Fibrosis

Male wild-type C57BL/6 mice (8–10 weeks, 18–22 g) were purchased from the Charles River (Beijing, China). Mice were housed in a specific pathogen-free room with a 12 h light/dark cycle, controlled temperature and humidity, and free access to food and water. For bleomycin administration, mice were anesthetized with isoflurane followed by intratracheal instillation of bleomycin (1.5 U/kg.bw, i.t.) or equivoluminal (50 μL) 0.9% saline, as previously described [44]. For drug intervention, regular chow or FBZ-impregnated chow (containing 150 ppm FBZ) were given ad libitum from day 10 after the bleomycin challenge. Daily chow consumption and mouse weight were recorded to calculate the dosage of FBZ administration. On the 21st day, the mice were sacrificed, and samples were collected.

### 4.3. Lung Histological Analysis and Immunohistochemical Staining

For histological analysis, whole lungs were fixed for 24 h in 4% phosphate-buffered formalin (PFA), dehydrated and embedded in paraffin, and 5 μM sections were stained using a Masson trichrome staining kit (G1340, Solarbio, Beijing, China) following the manufacturer’s protocols. To develop a modified Ashcroft score, 5 fields in each lung section were randomly selected under 100× magnification to obtain the mean score by two pathologists in a blinded fashion [45]. For immunohistochemical staining of a-SMA and collagen I, tissue slides were dewaxed by dimethyl benzene, polarized with descending concentrations of alcohol, and rinsed with deionized water. Endogenous peroxidase activity was blocked by incubating sections in 3% hydrogen peroxide solution at room temperature for 30 min after antigen retrieval. The 10% normal goat serum was used to block the slides for 1 h. Primary antibodies were incubated overnight at 4 °C followed by incubation of secondary antibody for 60 min at room temperature. The slides were visualized by DAB staining, and counterstaining was performed with hematoxylin. Images of the stained sections were obtained with a panoramic scanner (3DHISTECH, Budapest, Hungar).

### 4.4. Hydroxyproline

The hydroxyproline content of the right lung was determined by using a hydroxyproline assay kit (MAK008, Sigma, St. Louis, MO, USA) following the manufacturer’s protocols. Briefly, right mouse lungs were homogenized in 100 μL of water for every 10 mg of tissue. Next, isopycnic HCl (12 N) was added to the sample of homogenate. Samples in Teflon-capped vials were hydrolyzed at 120 °C for 3 h. Then, 10 μL of each sample was transferred to a 96-well plate and evaporated at 60 °C until dry. Chloramine T reagent (100 μL) was added to each sample and incubated at room temperature for 5 min. p-Dimethylaminobenzaldehyde reagent (100 μL) was added to each well and incubated for 90 min at 60 °C. Absorbance was measured (λ = 560 nm) using a BioTek ELx800 plate reader (Winooski, VT, USA). The amount of hydroxyproline in the samples was calculated using standard rat tail collagen, prepared according to the manufacturer’s protocol (Sigma, St. Louis, MO, USA).

### 4.5. Cell Culture

The human embryonic lung fibroblast cell line IMR-90 (purchased from Procell, Wuhan, China) was maintained in Dulbecco’s modified Eagle’s medium (DMEM, Hyclone, Logan, UT, USA) supplied with 10% fetal bovine serum (FBS), L-glutamine (2 mM), 100 IU penicillin, and 100 mg/mL streptomycin at 37 C in 5% CO_2_. All cell experiments were performed within 10 passages. For fibroblast stimulation, cells were switched to serum-free media overnight prior to TGF-β1 (5 ng/mL) addition.

### 4.6. CCK-8 and EdU Incorporation Assay

IMR-90 cells were seeded in 96-wells plates overnight followed by serum-starve for 24 h, then treated with FBZ at the indicated concentration for the indicated period. CCK-8 (BA00208, Bioss, Beijing, China) was added to the well and incubated for 2 h at 37 °C, and absorbance was measured (λ = 450 nm) using a BioTek ELx800 plate reader. EdU incorporation was performed using a Cell-Light EdU Apollo567 in vitro Kit (C10310-1, Ribobio, Guangzhou, China) following the manufacturer’s protocols. Images were obtained using an inverted fluorescence microscope (Nikon, Tokyo, Japan).

### 4.7. Determination of Glucose Concentration in Culture Medium

IMR-90 cells were seeded in a 96-well plate overnight followed by glucose starving in glucose-free DMEM medium (90113, Solarbio, Beijing, China) for 2 h. Culture medium was changed to complete medium with indicated concentration of FBZ or 50 μM apigenin as the positive control, and culture supernatants were collected at indicated times. Glucose concentration was determined by glucose oxidation assay using a GO assay kit (BC2505, Solarbio, Beijing, China) according to the manufacturer’s instructions.

### 4.8. Cell Cycle Analysis

IMR-90 cells were seeded in a 6-well plate overnight followed by serum-starve for 24 h, then treated with indicated compounds for 24 h. Cells were harvested followed by fixation in 75% EtOH. RNase and PI were added and incubated for 10 min at room temperature before the cell cycle was analyzed by flow cytometry (BD Biosciences, San Jose, CA, USA).

### 4.9. Immunofluorescence Staining

Cells were seeded on glass coverslips in a 24-well plate. After indicated treatments, cells were fixed with 4% paraformaldehyde in PBS for 30 min at room temperature. Cells were then washed with PBS and subsequently permeabilized with 0.3% Triton-100 in PBS for 10 min and then blocked with 10% goat serum in PBS for 1 h at room temperature. Cells were then incubated overnight at 4 °C with primary antibodies followed by incubation for 1 h at room temperature with Dylight 488- or Dylight 594-conjugated secondary antibodies. F-actin was stained with TRITC-Phalloidin (CA1610, Solarbio, Beijing, China), and cell nuclei were stained with DAPI (C0065, Solarbio, Beijing, China). Subsequently, the coverslips were removed from the wells and mounted on glass slides with anti-fading mounting medium (S2100, Solarbio, Beijing, China). Images were obtained using a laser scanning confocal microscope (Leica, Wetzlar, Germany).

### 4.10. Glycolytic Function Assay

Extracellular acidification rate (ECAR) as a parameter of glycolytic flux was measured on a Seahorse XF96 bioanalyzer (Agilent technologies, Santa Clara, CA, USA) using a XF Glycolysis Stress Test kit (103020-100, Agilent technologies, Santa Clara, CA, USA) according to the manufacturer’s instructions. Briefly, IMR-90 cells were seeded on a 96-well assay plate overnight, then starved in a serum-free medium for 24 h. The medium was changed to complete medium, then treated with or without active TGF-β1 (5 ng/mL) for 24 h. For FBZ treatment, FBZ at the indicated concentrations was added 4 h before tests and was maintained in the assay medium.

### 4.11. Western Blot Analysis

Western blot analyses were performed as described previously. Briefly, cell lysates or lung homogenates were prepared using RIPA lysis buffer (P0013B, Beyotime, Shanghai, China) supplied with protease and phosphatase inhibitors. The protein concentrations in the supernatants were determined using the BCA kit (Solarbio, Beijing, China) with BSA as standard. Samples containing equal quality of protein were subjected to SDS-PAGE and electrotransferred to PVDF membranes (Millipore, Darmstadt, Germany). The membranes were blocked in 5% skim milk, then incubated with specific primary antibodies overnight at 4 °C, followed by incubation with HRP-conjugated secondary antibodies for 1 h at room temperature. Bands were visualized using enhanced chemiluminescence (Thermol fisher, Waltham, MA, USA) and images were captured on an Odyssey Imaging System (LI-COR Biosciences, Lincoln, NE, USA). The densitometry band quantification was performed using the Image Studio software (LI-COR Biosciences, Lincoln, NE, USA).

### 4.12. Quantitative RT-PCR Analysis

Cells were lysed using TRIzol (Takara, Dalian, China) to extract total RNA. cDNA was transcribed using the GoScript™ Reverse Transcription System (A5001, Promega, Madison, WI, USA). The reaction was performed using the Quanti Nova SYBR Green PCR Kit (208052, QIAGEN, Hilden, Germany) on a LightCycler 480 system (Roche, Penzberg, Germany). Relative mRNA levels were quantified by calculating the comparative 2-ΔΔCt method. The following primers were used for each target gene: *ACTB* (5′-GGGAAATCGTGCGTGACAT-3′, 5′-CTCATTGCCAATGGTGATGA-3′); *ACTA2* (5′-CTCTGGACGCACAACTGGCATC-3′, 5′-CACGCTCAGCAGTAGTAACGAAGG-3′), COL1A1 (5′-GTGCTAAAGGTGCCAATGGT-3′, 5′-ACCAGGTTCACCGCTGTTAC-3′), and *FN1* (5′-CGTGTACCATCGCAAACCG-3′, 5′-ACCACATAGGAAGTCCCAGCA-3′).

### 4.13. Statistical Analysis

Comparisons between two experimental groups were carried out using two-tailed Student’s *t*-test. Multi-groups were compared using one-way or two-way analysis of variance with Tukey’s multiple comparison test. All experiments were conducted with at least 3 independent replications. The data are presented as the mean ± SEM/SD. In all cases, *p* < 0.05 was considered as statistical significance.

## 5. Conclusions

Our data suggested that FBZ has potential as a novel treatment of pulmonary fibrosis. The results also suggested that FBZ acts as a metabolic regulator in the treatment of other metabolism-related diseases. Further efforts are needed to investigate the molecular mechanism of the anti-fibrotic property of FBZ to facilitate its repurposing against fibrotic diseases.

## Data Availability

All data generated or analyzed during this study are included in this published article; further inquiries can be directed to the corresponding author.

## Supplementary Materials

The supporting information can be downloaded at: https://www.mdpi.com/article/10.3390/ijms232214088/s1.
