# Supplementary material for: Fenbendazole Attenuates Bleomycin-Induced Pulmonary Fibrosis in Mice via Suppression of Fibroblast-to-Myofibroblast Differentiation
Source: Int J Mol Sci. 2022 Nov 15;23(22):14088. doi: 10.3390/ijms232214088 (PMC9693227; doi:10.3390/ijms232214088)
Supplement: Supplementary file 1 [file ijms-23-14088-s001.zip › ijms-1992979-supplementary.pdf]

Supplementary Materials

Table S1. Daily Consumption of Chow and FBZ (Mean ± SD)

Groups	**Chow Daily Consumption （g/kg.bw）**	**FBZ Daily Intake (mg/kg.bw)**
Saline	152.82 ± 26.27	-
FBZ	165.60 ± 27.97	24.84 ± 4.20
BLM	138.51 ± 21.79	-
BLM+FBZ	147.12 ± 27.47	22.07 ± 4.12

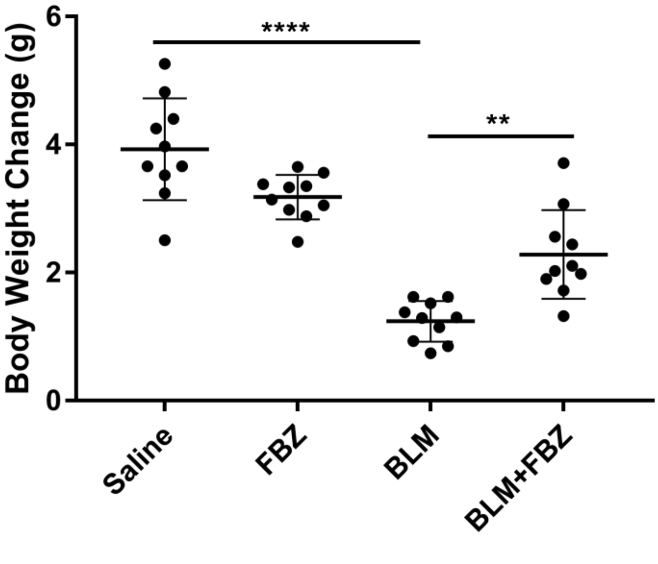

Figure S1. Endpoint body weight changes. **$P < 0.01$, and ****$P < 0.0001$.
